# Investigations of Ascorbic Acid Synthesis and Distribution in Broiler Tissues at Different Post-Hatch Days

**DOI:** 10.3390/life13051137

**Published:** 2023-05-06

**Authors:** Liping Gan, Yifeng Zhao, Peng Wang, Chenkai Zhao, Yilei Li, Weihao Huang, Liuying Shi, Yaoming Cui, Hanzhen Qiao, Jinrong Wang, Yuming Guo

**Affiliations:** 1 School of Bioengineering, Henan University of Technology, Zhengzhou 450001, China; lpgan@haut.edu.cn (L.G.);; 2 Department of Animal Science and Feed Technology, China Agricultural University, Beijing 100191, China

**Keywords:** broiler, ascorbic acid, L-gulonolcactone oxidase, SVCT1/2

## Abstract

Ascorbic acid (AA) is an indispensable nutrient required to sustain optimal poultry health and performance, which is commonly excluded from the diet of broilers. To investigate the synthesis and distribution of AA during broiler growth and clarify its possible turnover, 144 1 d old healthy Arbor Acres broilers with a body weight of approximately 41 g were randomly assigned to eight groups of 18 broilers each. The kidney, liver, ileum, and spleen of one bird from each group were collected every week until 42 d to detect the synthesis capacity, tissue distribution, and transporter gene expression of AA. The results showed that kidney L-gulonolactone oxidase (GLO) activity responded quadratically (*p* < 0.001), with maximum activity observed at 7 to 21 d old. Hepatic total AA and dehydroascrobate (DHA) concentration increased linearly (*p* < 0.001) with age, as did splenic total AA (*p* < 0.001). In the ileum, mRNA expression of sodium vitamin C transporter 1/2 (SVCT1/2) decreased with the growing age of the broilers (*p* < 0.05). The expression of SVCT1 in the kidney was not influenced by the growing age of the broilers. The progressive buildup of AA in the liver and spleen of broilers as they age implies an amplified demand for this nutrient. The waning synthesis capacity over time, however, raises concerns regarding the possible inadequacy of AA in the latter growth phase of broilers. The addition of AA to the broilers’ diet might have the potential to optimize their performance. However, the effectiveness of such dietary supplementation requires further investigation.

## 1. Introduction

Ascorbic acid (AA), commonly known as vitamin C, is a crucial water-soluble antioxidant that plays essential roles in maintaining the health and optimal functions of broilers. The antioxidant activity of AA stems from its ability to be reversibly oxidized to ascorbyl free radical and further to dehydroascrobate (DHA) [1]. Some species, such as humans, some passeriform birds, and guinea pigs can no longer synthesize AA due to a nonfunctional mutant L-gulonolactone oxidase (GLO) [2], and thus require dietary supplementation to meet their needs. In contrast, AA is usually excluded from the diet of broilers [3] because chickens can synthesize AA via the glucuronatexylulose cycle by GLO in the kidney [4,5]. The absorbed and/or synthesized AA can be transported to cells by sodium-dependent vitamin C transporters—specifically, SVCT1 and SVCT2. The function of SVCT1 as an AA transporter in chickens has been confirmed [6]. The tissue distribution of the two transporters is dependent on their properties; SVCT1 is primarily found in epithelial tissues, while SVCT2 is found mainly in tissues requiring adequate AA concentrations, such as brain and spleen [1]. In a previous study, the authors found that among the foregut segments of chickens, the ileum had the highest gene expression of SVCT1, and the kidney also had high gene expression of SVCT1 [5]. The expression of SVCT1 in the ileum is responsible for the absorption of AA from dietary sources, while SVCT1 in the kidney facilitates the reabsorption of AA from urine, both of which play vital roles in the tissue accumulation of AA.

The tissue concentration of AA relies on its requirements for this nutrient. The accumulated AA in tissues generally has three sources: synthesis de novo from glucose in the kidney (such as in chicken, etc.), or from the liver (such as in pigs, etc.), uptake AA via SVCT1/2 or DHA via glucose transporters from diet, and reabsorb AA in the kidney through SVCTs, chiefly SVCT1 [7]. The increased AA inside a cell could also stem from the reduction of DHA to AA [8]. In laying hens, unlike in the kidney and ovary, where the concentration of AA is super low (under 0.4 μmol/g weight), the concentration of AA in the liver and spleen is as high as in the brain (more than 1.5 μmol/g weight) [5]. The AA inside the liver and spleen plays a vital role in maintaining their physiological function. As an antioxidant, AA could neutralize free radicals that are generated during the normal metabolic processes of the liver and spleen, thus protecting them from oxidative stress and damage [9]. Furthermore, AA acts as a cofactor of the enzymes involved in collagen synthesis, thereby preserving the structure and function of the liver and spleen. Additionally, AA enhances the differentiation and proliferation of B- and T lymphocytes and suppresses inflammation by inhibiting the production of inflammatory mediators via the inhibition of enzymes such as cyclooxygenase and lipoxygenase [10]. It is vital to monitor the concentration of AA in the liver and spleen to better understand its critical functions in broilers.

The inclusion of AA in the diet of broilers is typically unnecessary due to their ability to synthesize it. The ability of broilers to synthesize AA in the kidney can be affected by various factors, such as gender, feed supply, and additives [4,11,12]. The adequacy of AA for broilers under different conditions and in different physical states has also been questioned. Numerous studies have focused on the effects of supplemental AA in the diet of broilers on the antioxidant system, immunity, hatchability, and performance. The conclusions, however, have been inconsistent. While some researchers argue that exogenous AA has no significant impacts on production performance [13], others have reported enhanced body weight and increased immunity in broilers given additional AA [14,15,16,17]. The inconsistent results have prompted investigations into the factors affecting GLO activity and AA synthesis ability [4]. It is important to quantify GLO activity and AA concentration in tissues, particularly in the liver and spleen of broilers, along with assessing the expression of AA transporters. The deposition amount of AA in tissues has significance for clarifying the requirement of AA during the feeding period of broilers. Additionally, the study of the expression of its transporter can not only clarify the mechanism of its absorption and transport, but also indirectly provide a theoretical basis for whether exogenous supplementation of AA is needed. Thus, the purpose of this experiment is to investigate the changes in GLO activity at different ages of birds during the feeding period, the distribution of AA in liver and spleen, and the gene expression of AA transporters in ileum and kidney, to provide a theoretical basis for the application of AA in broiler production.

## 2. Materials and Methods

### 2.1. Animals, Management, and Sampling

All experimental procedures including animal experimental protocols, management, and sampling management conditions were approved by the Animal Care and Use Committee of China Agricultural University. A total of 144 1 d old male Arbor Acres broilers, with similar health statuses, were allocated to 8 battery cages, with 18 birds in each cage. All birds have free access to the same standard commercial feed and tap water. The ingredient and nutrient composition of the feed is presented in Table 1. The birds were maintained in a room where the temperature and the humility are controllable. The light scheme applied in the experiment was a 20 h light and 4 h dark cycle. The birds were vaccinated using Newcastle disease virus and infectious bronchitis virus at d 9 and infectious bursal disease virus at d 21 via intranasal and intraocular method. The temperature was maintained at 33~35 °C on the day of the birds’ arrival and on day 2, and was gradually reduced by 1 °C each day until it reached 21 °C on d 21 and thereafter. All the animal management procedures employed in this experiment were based on the recommendations of Arbor Acres management handbooks.

On d 1, 2 broilers were randomly chosen from each cage, while 1 broiler was randomly picked from each cage at d 7, 14, 21, 28, 35, and 42, and sacrificed by intravenous administration of pentobarbital anesthesia at a dose of 50 mg/kg body weight. Then, the body cavity was immediately opened to collect the liver, the spleen, the kidney, and the ileum. On d 1, the whole liver, kidney, and spleen were collected, while from d 7 to 42, the whole spleen, the left lobe of the liver, and the middle part of the left kidney were collected from broilers. All the samples were covered with aluminum foil, snap frozen in liquid nitrogen, and preserved in −80 °C until further analysis.

### 2.2. Measurement of L-gulonolactone Oxidase Activity in Kidneys

The AA synthesis ability of broilers at different ages was determined by evaluating the GLO enzyme activity in the kidney. The method used to measure GLO activity was based on the rate of total AA (both AA and DHA) synthesized in the kidney tissue by adding L-gulonolactone [18]. The detailed procedure was described before [5]. Briefly, approximately 100 mg of kidney was homogenized using sodium phosphate buffer with 0.2% sodium deoxycholate (TCI, Shanghai, China), followed by centrifuging at 20,000× *g* for 30 min at 4 °C. Then, the supernatants were added with L-gulonolactone to bring its final concentration to 5 mmol/L and were incubated in water for 30 min in the dark. A blank without L-gulonolactone was run to correct the endogenous AA. After 30 min incubating, the reaction was stopped by adding the same volume of 5% trichloroacetic acid, followed by 20 min incubating at room temperature in the dark, after which the mixture was centrifuged at 4 °C, 10,000× *g* for 5 min. The supernatant was used to detect GLO-synthesized AA.

### 2.3. Ascorbic Acid Tissue Deposition

The HPLC with UV light detector was employed to determine the AA concentration in the liver and spleens of broilers at different ages. Briefly, approximately 120 mg livers or spleens were homogenized in 1.6 mL cold metaphosphoric acid solutions and centrifuged at 16,000× *g* for 15 min at 4 °C. The obtained supernatants were used to measure the total AA and AA levels. For the total AA measurement, an equal volume of 5 mmol/L Tris (2-carboxy ethyl) phosphine hydrochloride (TCEP) in distilled water (pH = 2) was added and allowed to react for 2 h at 4 °C in the dark. To detect the AA concentration in tissues, an equal volume of distilled water was added instead of TCEP and maintained in the dark at 4 °C for 2 h. After incubating for 2 h, the mixture was centrifuged at 12,000× *g* for 10 min at 4 °C, about 10 μL of the supernatant was applied to run onto the HPLC column for AA detection. The HPLC system was purchased from Waters, equipped with Waters 717 plus autosampler and Waters 2487 dual wavelength UV detector. The column and the mobile phase used in this study was described before [5]. Finally, the DHA levels in tissues were calculated by using the total AA minus the AA levels in the liver and spleen.

### 2.4. Quantitative Real-Time PCR

Total RNA was extracted from ileum and kidney using TRIzol reagent (TAKARA Bio., Beijing, China) according to the manufacturer’s instructions. A nanodrop 2000 was applied to quantify the RNA while the gel electrophoresis was employed to check the RNA integrity. The PrimeScript RT reagent kit with gDNA eraser (TaKaRa, Dalian, China) was used for the reverse transcription according to the manufacturer’s instructions. Quantitative real-time PCR (qRT-PCR) was performed in triplicate on an Applied Biosystems 7500 Fast Real-Time PCR System (Thermo Fisher Scientific, Waltham, MA, USA) with the TB Green Premix Ex Taq (TaKaRa, Dalian, China). The primes sequences of SCVT1, SCVT2, GLO, and GAPDH used in the present experiment was shown in Table 2. The transcript amplification results were analyzed with the ABI 7500 software v2.3 and all values of different genes were normalized to the expression of housekeeping gene GAPDH using comparative 2^−ΔΔCt^ method according to Livak and Schmittgen (2001) [19].

### 2.5. Statistical Analysis

The statistical analysis of the data was performed using SPSS statistical software (SPSS for Windows, version 22.0, SPSS Inc., Chicago, IL, USA). One-way analysis of variance was conducted, followed by Tukey’s multiple comparisons, to compare the differences among the different ages of broilers. Linear and quadratic contrasts were utilized to evaluate the statistical differences of AA concentrations, gene expression of SVCT1/2, and AA synthesis ability of broilers during the 1–42 d growth period. A statistically significant difference was defined as *p* ≤ 0.05.

## 3. Results

### 3.1. GLO Enzyme Activities

**Figure 1:**
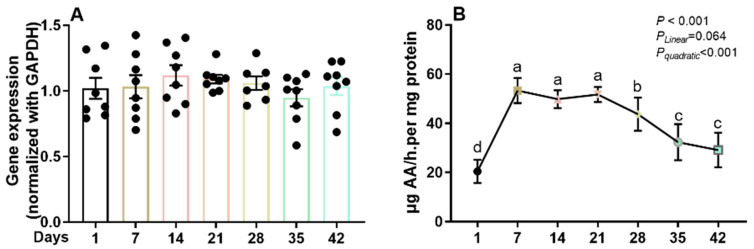
The gene expression (**A**) and enzyme activity (**B**) of L-gulonolactone oxidase (GLO) in the kidneys of broilers at different ages were measured. Within each panel, means without a common letter differ significantly at *p* < 0.05.

GLO is responsible for the final step in the metabolic pathway that produces ascorbate in animals. As shown in Figure 1A, the gene expression of GLO in the kidneys of the broilers remained consistent from d 1 to d 42. However, the GLO enzyme activities differed significantly during the 42-day growth period (*p* < 0.001). In addition, the GLO enzyme activities in broiler kidneys showed quadratic effects (*p* < 0.001), with the lowest enzyme activity occurring at d 1 and the highest enzyme activities observed from d 7 to d 21. Subsequently, the GLO enzyme activity decreased gradually in broiler kidneys.

### 3.2. The Concentration of AA in Liver and Spleen of Broilers

**Figure 2:**
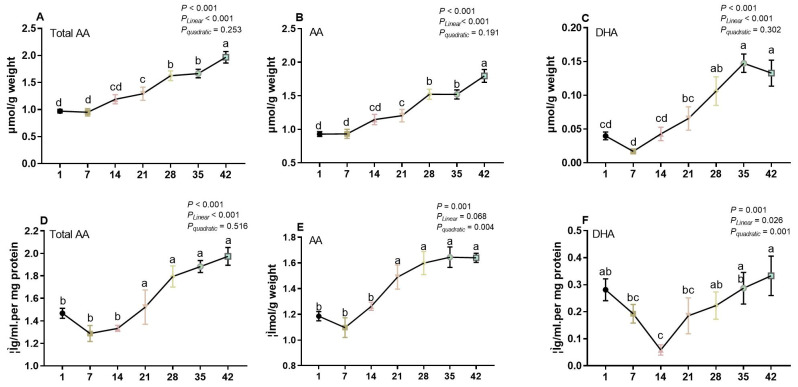
The concentration of total ascorbic acid (AA), AA, and dehydroascorbic acid (DHA) in the liver (panels **A**–**C**) and spleen (panels **D**–**F**) of broilers at different ages were analyzed. In each panel, means that do not share a common letter are significantly different at *p* < 0.05.

Different body tissues have varying concentrations of AA, and those with high metabolic needs generally have greater concentrations. In a previous study, the authors identified spleen, brain, and liver as poultry tissues with high AA concentrations (ranging from 1.2 to 2.0 μmol/g wet tissue), while kidney, shell gland, and ovary had lower concentrations (ranging from 0.1 to 0.4 μmol/g wet tissue) [5]. In this study, the liver and spleen AA concentrations of broilers were measured at different ages. As shown in Figure 2A–C, the concentrations of AA, DHA, and total AA in the liver increased linearly with broiler age (*p* < 0.001). From d 1 to d 14, the levels of AA, DHA, and total AA remained unchanged, while from d 21 to d 42, they continued to increase until reaching the highest level at d 42. DHA accumulation followed a similar pattern, with the highest level at d 35 and d 42. In the spleen, the levels of total AA increased linearly with broiler age (Figure 2D, *p* < 0.001). Although significant linear trends were observed in splenic total AA concentrations, no significant differences were found between d 1, d 7, and d 14, or between days 21, 28, 35, and 42 (*p* > 0.05). The splenic AA concentrations were higher from d 21 to 42 and showed a positive quadratic response (*p* < 0.001) to broiler age. A quadratic effect was also observed for splenic DHA level over the 42 days growth period, with the lowest level seen at d 14 and the highest at d 42 (*p* < 0.001, Figure 2F).

### 3.3. The Gene Expression Levels of SVCTs in Ileum and Kidney of Broilers

**Figure 3:**
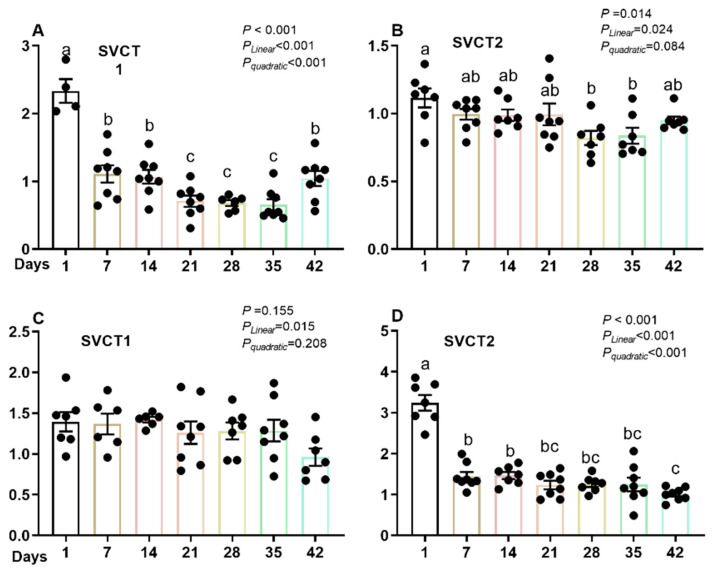
The gene expression of SVCT1 in the ileum (**A**) and kidney (**C**), and SVCT2 in the ileum (**B**) and kidney (**D**) of broilers at different ages was analyzed. SVCT refers to the sodium vitamin C dependent transporter. Means within each panel that do not share a common letter differ significantly at *p* < 0.05.

The gene expression of SVCTs in ileum was analyzed to detect the AA absorption ability during the growing period. As shown in Figure 3A,B, SVCT1 expression in the ileum was significantly affected by age. As age increased, the SVCT1 expression level declined erratically, showing both significant linear and quadratic trends (*p* < 0.001). The highest expression of SVCT1 in ileum occurred at d 1. After d 1, the expression of SVCT1 in ileum dropped markedly to less than half of that at d 1, and the lowest expressions were observed from d 21 to 35. As for the expression of SVCT2, during the 42-day growing period, the SCVT2 gene expression decreased slightly in a linear fashion with increased age (*p* = 0.024) but approached a quadratic response (*p* = 0.087). Similar to SVCT1, the expression of SVCT2 in the ileum also showed the highest level on d 1 (Figure 3B).

Ascorbic acid can be reabsorbed by kidney tubular cells through SVCTs, which is also an important way for animals to collect the AA needed by the body. In broilers, the gene expression of SVCT1 in kidneys showed a linear trend (*p* = 0.015) with increasing age. However, the SVCT2 expression exhibited a significant difference due to different ages (*p* < 0.001) and showed linear and quadratic trends to growing age (both *p* < 0.001). The gene expression of SVCT2 in kidneys was highest on d 1 (Figure 3D).

## 4. Discussion

As chickens are capable of synthesizing AA in their kidneys, the nutrient is typically excluded from the diets of broilers under normal conditions. Consequently, broiler tissues accumulate AA primarily through three physiological pathways, including de novo biosynthesis from glucose in the kidney, reabsorption from urine via SVCT1 in nephrocytes, and reduction from cellular DHA [8,20]. L-gulonolactone oxidase is an enzyme that is essential for the final step of AA synthesis in animals’ tissues. The lack of GLO leads to biosynthesis failure, and these animals solely rely on dietary AA supplementation to fulfill their AA requirements. Nonetheless, the activity of GLO can be influenced by various factors, including thermal stress and other stressors. Under stressful conditions, the activity of GLO may be inhibited, resulting in a reduced amount of synthesized AA. This observation justifies the need for AA supplementation in heat-stressed broilers [21]. In the current study, the trend of GLO enzyme activity in broilers from 1 to 42 d of age is consistent with previous research on rats [22], which showed an increase in activity after birth followed by a decline with age. Nevertheless, the present results differ from those reported by Hooper, Maurice, Lightsey, and Toler [4] who observed maximum GLO activity at 13 days, followed by a decline to mature levels. These discrepancies may be due to differences in the method of AA detection used. The HPLC procedure utilized in this experiment was more sensitive and accurate for detecting low levels of AA compared to the spectrophotometric analysis, resulting in more reliable results that reflect the dynamic of AA synthesis in broilers. In addition, it is worth noting that chicken embryos have the capacity to synthesize AA in the kidney (metanephros and mesonephros) and yolk sac membrane, with synthesis rates increasing with the development stage [23,24]. The high biosynthesis rate of broilers at hatching explains their high need for AA, as they grow rapidly and are exposed to a complex environment that requires a greater amount of AA to cope with. However, GLO catalyzes the conversion of L-gulonolactone to AA with the production of H_2_O_2_, which can result in glutathione depletion and be detrimental to broilers [8]. As the broilers grow, the antioxidant system also develops, which may rely less on the AA synthesis since the formation of H_2_O_2_ requires extra antioxidants in the body. This may partly explain the decline in GLO enzyme activity after 21 days.

Ascorbic acid can be transported to cells through SVCTs. The ileum, rather than duodenum or jejunum, is the primary site for chickens’ absorption of AA from their diet [5]. In this experiment, the gene expression of both SVCT1 and SVCT2 in the ileum declined as the broilers grew, which is consistent with the trend observed for SVCT1 in rat liver [25]. It has been reported that the feedback mechanisms exist to regulate the abundance of ascorbate transporters at the cell surface, with important consequences for the modulation of ascorbate absorption by enterocytes, reabsorption by kidney tubular cells, and uptake into target cells. The decreased gene expression of SVCTs in the present experiment may suggest the decreased ability of cells to absorb AA. This may be due to the lack of AA in the diet of the broilers in the experiment, as the synthesis of SVCTs is not needed when there is little AA in the diet to be absorbed. As noted, the expression of both SVCT1 and SVCT2 was highest on d 1, which implies that young broilers might require more AA. It also may be concluded that supplementing the diet with AA during the early stages of a broiler’s life may result in a higher absorptive capacity. It has been reported that SVCT1 in the kidney plays a vital role in renal AA absorption from urine. Loss of SVCT1 in mice leads to a loss of as much as 70% of their ascorbate body stores through daily urination [26]. The expression of SVCT1 showed no differences during the 42-day growth period of broilers in the kidney, which implied unchanged reabsorption of AA from urine. However, the expression of SVCT2 in the kidney declined markedly, which to some extent demonstrates a decline in urinal AA as the broilers grew. It has been reported that in mice, the urinal AA increased significantly from 3 months to 6 months of age but decreased markedly thereafter to almost undetectable levels at 30 months of age [27]. The urinary AA contents may reflect the AA requirements of animals. When their need for AA cannot be satisfied, they may reabsorb more AA to prevent any loss of AA in urine. However, it is not easy to collect urine from chickens to detect AA concentrations. Therefore, the expression of SVCTs may reflect the urinary AA concentrations in broilers. The decreased expression of SVCT2 in broiler kidneys from d 1 to d 42 may reflect the slightly decreased AA concentration in urine, which also demonstrates a higher requirement for AA after hatchery.

Ascorbic acid participates in a number of reactions required for normal cell functions, and its accumulation reflects the requirements of different tissues. Ascorbic acid acts as an electron donor and can be easily oxidized, which explains its powerful antioxidant capacity. As a coenzyme factor, AA also plays crucial roles in a lot of physiological activities in animals. In chickens, the liver is one of the tissues with the most abundant levels of AA [5]. It is well known that the liver has a central role in whole-body homeostasis, and is responsible for metabolism, synthesis, storage, and redistribution of nutrients [28]. Sublethal endotoxin was found to increase ascorbate recycling and ascorbate concentration in the liver to protect it against the reactive oxygen species produced by all the challenges [7]. Similarly, partial hepatectomy also caused an increased level of hepatic ascorbate [28]. In the present experiment, the concentration of AA increased with the age of the broilers, indicating a growing requirement for AA with age. The synthesis ability of AA, however, decreased after 21 days of age. Therefore, the increased AA in livers might result from AA recycling at the expense of glutamine and AA reabsorption by the kidney. Ascorbate also contributes to immune defense by supporting various cellular functions of both the innate and adaptive immune system. Ascorbic acid could promote the maturation of T cells, as well as the proliferation and differentiation of B cells. Moreover, the macrophage phenotype and function can be enhanced by supplementing with AA in AA-deficient mice [10,29,30,31]. The spleen combines the innate and adaptive immune system in a unique manner [32] and plays vital roles in the immune system of chickens. In both mammals and chickens, one of the highest levels of AA concentration is in the spleen. Consistent with previous studies on mice, the levels of AA in broiler spleens increase with growing age [27]. The increased concentration of AA in broiler spleens could maintain the physical functions of the spleen and enhance broiler immunity. The increased AA levels in both livers and spleens indicate the increasing requirement of AA in broilers as they grow. In ovo injection of AA has been reported to have positive effects on posthatch growth and the antioxidant capacity of broilers [24,33]. A previous study also indicated that supplementing broilers’ diets with AA induced lower GLO enzyme activities while increasing concentrations of AA in livers and spleen, resulting in better production performance and enhanced antioxidant ability [14]. The dietary inclusion of AA has been shown to increase its concentration in tissues and inhibit de novo synthesis in the kidney, thus indicating the ability of broilers to absorb AA from their diet and potentially impacting the feedback loop of AA synthesis in the body. As previously discussed, the de novo synthesis of AA is dependent on glucose and produces H_2_O_2_, which requires the presence of antioxidants to be removed from the body. Therefore, including AA in the diet of broilers may yield more favorable results compared to relying solely on their endogenous synthesis.

## 5. Conclusions

The results of GLO gene expression and enzyme activity suggest that gene expression may not always accurately reflect protein function, and future studies should focus on detecting the protein levels of SVCT1/2 in broilers. The research found that there is a decrease in AA synthesis during the late phase of the broiler growing period, while the concentration of AA in their tissues increases. This implies that the endogenous synthesis of AA may not fully meet the birds’ needs during the developmental stage. The study also suggests that inhibiting the energetically costly process of de novo synthesis of AA may have benefits for broiler production. Nevertheless, further studies are necessary to determine whether exogenous AA supplementation can optimize the birds’ production performance.

## Figures and Tables

**Table 1:** Composition and nutrient contents of the diet used in the study.

Item, %	0~3 Weeks	4~6 Weeks
Corn	53.82	59.76
Soybean meal	38.54	33.22
Soybean oil	3.52	3.5
Calcium carbonate	1.76	1.21
Calcium phosphate	1.26	1.39
Sodium chloride	0.35	0.3
Choline chloride (50%)	0.2	0.2
L-Lys × HCl (78%)	0.2	0.16
DL-Met (98%)	0.15	0.1
Mineral premix ^1^	0.18	0.13
Vitamin premix ^2^	0.02	0.02
Total	100	100
Nutrient Level ^3^		
AME, MJ/kg	12.36	12.6
Crude protein, %	22.02	19.72
Calcium, %	1	0.9
Available phosphorus, %	0.45	0.35
Lysine, %	1.19	1.04
Methionine, %	0.5	0.43
Methionine + Cystine, %	0.85	0.75

^1^ Provided per kilogram of diet: Cu, 8 mg; Zn, 75 mg; Fe, 80 mg; Mn,100 mg; Se, 0.15 mg; I, 0.35 mg. ^2^ Provided per kilogram of diet: vitamin A, 10,000 IU; vitamin D3, 2400 IU; vitamin E, 40 IU; vitamin K3, 2 mg; vitamin B1, 2 mg; vitamin B2, 6.4 mg; vitamin B6, 3 mg; vitamin B12, 0.02 mg; folic acid, 1 mg; niacin, 30 mg; Capantothenate acid, 10 mg. ^3^ Calculated.

**Table 2:** Primer sequences of housekeeping and target genes.

Gene Name		Primer Sequence ^1^, 5′-3′	NCBI Number	Product Size
GAPDH	F ^2^	GACCCCAGCAACATCAAATG	NM_204305.1	110 bp
	R	TTAGCACCACCCTTCAGATG		
GLO	F	TCTCCTCTGGATCAGCACCT	XM_015285218.1	131 bp
	R	AGCGGCACTCGTAGTTGAAG		
SVCT1	F	GGGATACCCACGGTGACCTC	XM_004944768.2	100 bp
	R	GCCGTGCACAGGAGTAGTAA		
SVCT2	F	TGTCTTGTGCTCCTCCTCCT	NM_001145227.1	101 bp
	R	TCCATTCCCTGTCCCAAATA		

^1^ Primer sequences are displayed in the 5′-3′ direction. ^2^ F, forward primer; R, reverse primer.

## Data Availability

The data used in this study are presented in the results section.

## References

[1] Figueroa-Méndez R., Rivas-Arancibia S. (2015). Vitamin C in Health and Disease: Its Role in the Metabolism of Cells and Redox State in the Brain. Front. Physiol..

[2] Young J.I., Zuchner S., Wang G. (2015). Regulation of the Epigenome by Vitamin C. Annu. Rev. Nutr..

[3] Council N.R. (1994). Nutrient Requirements of Poultry.

[4] Hooper C.L., Maurice D.V., Lightsey S.F., Toler J.E. (2000). Factors affecting ascorbic acid biosynthesis in chickens. I. Adaptation of an assay and the effect of age, sex, and food deprivation. J. Anim. Physiol. Anim. Nutr..

[5] Gan L., Fan H., Nie W., Guo Y. (2018). Ascorbic acid synthesis and transportation capacity in old laying hens and the effects of dietary supplementation with ascorbic acid. J. Anim. Sci. Biotechnol..

[6] Johnston L., Laverty G. (2007). Vitamin C transport and SVCT1 transporter expression in chick renal proximal tubule cells in culture. Comp. Biochem. Physiol. A Mol. Integr. Physiol..

[7] Kuo S.-M., Tan C.-H., Dragan M., Wilson J.X. (2005). Endotoxin Increases Ascorbate Recycling and Concentration in Mouse Liver. J. Nutr..

[8] Linster C.L., Van Schaftingen E. (2007). Vitamin C Biosynthesis, recycling and degradation in mammals. FEBS J..

[9] Kim J.H., Kang J.C. (2023). Detoxification effects of ascorbic acid on the oxidative stress, neurotoxicity, and metallothionein (MT) gene expression in juvenile rockfish, Sebastes schlegelii by the dietary chromium exposure. Fish Shellfish Immunol..

[10] Carr A.C., Maggini S. (2017). Vitamin C and Immune Function. Nutrients.

[11] Maurice D.V., Lightsey S.F., Abudabos A., Toler J.E. (2002). Factors affecting ascorbic acid biosynthesis in chickens. III. Effectof dietary fluoride on L-gulonolactone oxidase activity and tissue ascorbic acid (AsA) concentration. J. Anim. Physiol. Anim. Nutr..

[12] Maurice D.V., Lightsey S.F. (2007). Sexual difference in ascorbic acid synthesis, tissue ascorbic acid and plasma total antioxidant capacity in mature chickens. Br. Poult. Sci..

[13] Saki A.A., Rahmati M.M.H., Zamani P., Zaboli K., Matin H.R.H. (2010). Can Vitamin C Elevate Laying Hen Performance, Egg and Plasma Characteristics Under Normal Environmental Temperature?. Ital. J. Anim. Sci..

[14] Gan L., Fan H., Mahmood T., Guo Y. (2020). Dietary supplementation with vitamin C ameliorates the adverse effects of Salmonella Enteritidis-challenge in broilers by shaping intestinal microbiota. Poult. Sci..

[15] Mbajiorgu C.A. (2011). Effect of dietary ascorbic acid supplementation on egg production, egg quality and hatchability of indignous Venda chicken hens. Asian J. Anim. Vet. Adv..

[16] Cheng T.K., Coon C.N., Hamre M.L. (1990). Effect of environmental stress on the ascorbic acid requirement of laying hens. Poult. Sci..

[17] Torki M., Zangeneh S., Habibian M. (2013). Performance, Egg Quality Traits, and Serum Metabolite Concentrations of Laying Hens Affected by Dietary Supplemental Chromium Picolinate and Vitamin C Under a Heat-Stress Condition. Biol. Trace Elem. Res..

[18] Ching S., Mahan D.C., Moreau R.é., Dabrowski K. (2003). Modification of analytical procedures for determining vitamin C enzyme (L-gulonolactone oxidase) activity in swine liver. J. Nutr. Biochem..

[19] Livak K.J., Schmittgen T.D. (2001). Analysis of relative gene expression data using real-time quantitative PCR and the 2(-Delta Delta C(T)) Method. Methods.

[20] Mahan D.C., Ching S., Dabrowski K. (2004). Developmental aspects and factors influencing the synthesis and status of ascorbic Acid in the pig. Annu. Rev. Nutr..

[21] Abidin Z., Khatoon A. (2013). Heat stress in poultry and the beneficial effects of ascorbic acid (vitamin C) supplementation during periods of heat stress. Worlds Poult. Sci. J..

[22] Chatterjee I.B., Mckee R.W. (1965). Biosynthesis of l-ascorbic acid in rat liver microsomes: Influences of age, sex, dietary changes, and whole-body X-irradiation. Arch. Biochem. Biophys..

[23] Yew M.S. (1985). Biosynthesis of ascorbic acid in chick embryos. Experientia.

[24] Zhu Y., Zhao J., Wang C., Zhang F., Huang X., Ren Z., Yang X., Liu Y., Yang X. (2021). Exploring the effectiveness of in ovo feeding of vitamin C based on the embryonic vitamin C synthesis and absorption in broiler chickens. J. Anim. Sci. Biotechnol..

[25] Alexander J.M., Neha J., Hagen T.M. (2003). Age-related decline of sodium-dependent ascorbic acid transport in isolated rat hepatocytes. Arch. Biochem. Biophys..

[26] Corpe C.P., Tu H., Eck P., Wang J., Faulhaber-Walter R., Schnermann J., Margolis S., Padayatty S., Sun H., Wang Y. (2010). Vitamin C transporter Slc23a1 links renal reabsorption, vitamin C tissue accumulation, and perinatal survival in mice. J. Clin. Investig..

[27] Iwama M., Amano A., Shimokado K., Maruyama N., Ishigami A. (2012). The content of ascorbic acid in plasma and in 14 tissues of lambs of different ages during normal feeding and feeding restricted from time to time during the suckling period. J. Nutr. Sci. Vitaminol..

[28] Maldonado M., Inostroza E., Pena E., Moncada N., Mardones L., Medina J.L., Munoz A., Gatica M., Villagran M., Escobar E. (2017). Sustained blockade of ascorbic acid transport associated with marked SVCT1 loss in rat hepatocytes containing increased ascorbic acid levels after partial hepatectomy. Free Radic. Biol. Med..

[29] Mohammed B.M., Fisher B.J., Huynh Q.K., Wijesinghe D.S., Chalfant C.E., Brophy D.F., Fowler A.A., Natarajan R. (2014). Resolution of sterile inflammation: Role for vitamin C. Mediat. Inflamm..

[30] Manning J., Mitchell B., Appadurai D.A., Shakya A., Pierce L.J., Wang H., Nganga V., Swanson P.C., May J.M., Tantin D. (2013). Vitamin C promotes maturation of T-cells. Antioxid. Redox Signal..

[31] May J.M., Li L., Qu Z.C., Huang J. (2005). Ascorbate uptake and antioxidant function in peritoneal macrophages. Arch. Biochem. Biophys..

[32] Mebius R.E., Kraal G. (2005). Structure and function of the spleen. Nat. Rev. Immunol..

[33] Zhang H., Elliott K.E.C., Durojaye O.A., Fatemi S.A., Schilling M.W., Peebles E.D. (2019). Effects of in ovo injection of L-ascorbic acid on growth performance, carcass composition, plasma antioxidant capacity, and meat quality in broiler chickens. Poult. Sci..

